# Strength Parameters of Foamed Geopolymer Reinforced with GFRP Mesh

**DOI:** 10.3390/ma14030689

**Published:** 2021-02-02

**Authors:** Rafał Krzywoń, Szymon Dawczyński

**Affiliations:** Faculty of Civil Engineering, Silesian University of Technology, 44-100 Gliwice, Poland; szymon.dawczynski@polsl.pl

**Keywords:** foamed geopolymer, reinforcing lightweight concrete, glass fiber composites

## Abstract

The foaming of geopolymers lowers their density, thus opening up new environmental benefits, including acoustic and thermal insulation. At the same time, foaming disturbs the homogeneity of the material, which worsens the strength parameters, and particularly those related to tension, which can be improved by introducing reinforcement. This paper presents the results of research on foamed geopolymers reinforced with glass fiber meshes, a type of reinforcement that provides an adequate bond. The samples tested here were based on three types of coal fly ash, and were foamed with varying doses of hydrogen peroxide. Samples were cured at 40 °C and were tested after 28 days of maturing at ambient temperature. The strength parameters of the synthesized geopolymers were determined via laboratory testing, and were used to evaluate load-bearing capacity models of the tested samples reinforced with glass fiber mesh. The results showed the importance of the type of ash on the strength properties and efficiency of reinforcement. At the same time, a slight deterioration in the glass fibers was noticed; this was caused by the presence of sodium hydroxide solution, which was used as an activator.

## 1. Introduction

Coal fly ashes are valuable raw materials for the building materials industry, and especially for the manufacturing of cement and concrete. As a result of the transformation of the energy industry, new types of ashes are now being produced; these are mixtures of products from simultaneous coal combustion and gas desulphurization processes (ashes from fluidized bed boilers). They often contain high amounts of SO_3_ and CaO from unburned coal. For these reasons, they are sometimes treated as waste that is unsuitable for use in traditional cement production technologies. It is therefore necessary to look for other applications for these materials, one of which may be the synthesis of geopolymers.

Due to its significant content of silicon and aluminum, fly ash is an attractive material for use in the synthesis of geopolymers [1,2]. The traditional precursor, based on metakaolin, can be effectively replaced by fly ash of type F. The usefulness of fly ash in the synthesis of geopolymers is determined by its content of silicon and aluminum, since the Si/Al ratio determines the formation of a desirable type of zeolite. According to Tanaka et al. [3], a ratio of 0.9 gives a material that can be identified as a single-phase Na–A zeolite. This material is also produced at a lower rate at a ratio of around 1.7, and its crystallinity increases sharply at a ratio of 4.3.

Many studies have proved the superb strength properties of fly ash-based GPC (geopolymer cement) [4,5,6], which are comparable to those of OPC (ordinary Portland cement). They also provide good durability, and typically have better sulfate [7] and acid resistance [4,8], and excellent fire resistance [9]. Since it is cured at high temperatures, GPC has acceptably low shrinkage [10].

Another factor that may affect the properties of this material is the presence of calcium. In early studies, a higher amount of calcium (above 20%) was recognized as a contaminant, which caused a decrease in strength [11] and reduced the rate of geopolymer synthesis [12], and which produced various hydrates [13,14]. Recent studies, however, have highlighted other features with a beneficial effect on the properties of fresh mixture and hardened geopolymer [15]. The simultaneous formation of calcium silicate hydrate compounds in addition to aluminosilicate products under certain conditions improves the strength properties [16]; one example would be synthesis at ambient temperature [17,18]. Although thermal curing can still improve the properties of lignite coal fly ash [19,20], its properties are still worse than those of low-calcium Class F geopolymer [18].

The entrapment of air in the structure of a foamed geopolymer improves its thermal and sound insulating properties [21,22,23]. According to Cui et al. [24], foamed geopolymers may have better thermal and mechanical properties than foamed OPC. The lower density means that the weight of the structure is reduced, which allows for an additional reserve of load capacity. Another important benefit is the increase in compressive strength after exposure to elevated temperatures, a phenomenon that can be explained as an effect of geopolymer polycondensation and sintering at high temperatures [25]. Furthermore, the pores provide space to counteract the damage by heat [26]. Geopolymer foams can resist temperatures of up to 1000 °C without decomposition, and their shapes do not suffer dimensional changes within a temperature range of 400–800 °C [27]. These features mean that foamed geopolymers are very suitable for industrial applications as refractories, including furnace insulation and chimney cladding [28].

The main negative effect of foaming geopolymers is the reduction in the strength parameters. This problem affects most lightweight materials, and results from the disturbance in the homogeneous structure due to the presence of pores [29,30]. The relationship between the abovementioned advantages of foaming and strength reduction always involves a compromise, and the optimum parameters can be found by the proper addition of a foaming agent. Among the most common foaming techniques are the entrapment of air during mixing with surfactants (organic and inorganic technical foams) [31], and chemical foaming by the addition of peroxides such as hydrogen peroxide [32], sodium perborate [23], sodium hypochlorite [33] and the alkaline oxidation of metals, such as zinc, metallic silicon or aluminum [22,34,35].

Foamed geopolymers are brittle, and the ratio of the tensile to compressive strength, although better than for OPC concretes, is still very unfavorable. One remedy for this disadvantage may be the use of reinforcement. The most frequently studied method of reinforcing a geopolymer is the introduction of dispersed fibers to its structure, and various types of fibers have been investigated, including natural fibers (e.g., hemp [36], abaca [37]), and both organic (e.g., PVA [38], PP [39]) and inorganic high-strength fibers (e.g., glass [40], basalt [35,41] and carbon [42]). The most important limitation on the efficiency of reinforcement of foamed materials is the bond. In solid concrete, a suitable bond is ensured by chemical adhesion between the cement paste and steel, as well as the mechanical interlocking between the ribbed surface of the rebar and aggregate particles. The contact surface in porous materials is limited by the presence of voids, and flat fiber reinforcement does not usually provide interlocking conditions. The low thickness of the walls separating the pores and the brittleness of cellular concrete causes crushing of the local contact with the rebar rib. Although there has been no research on the reinforcement of foamed geopolymers with steel rebars, a study of this type of reinforcement in foamed OPC concrete shows that at a density of 1200 kg/m^3^, the strength of the bond is reduced by a factor of eight [43]. According to the authors of this study, the effectiveness of the bond in foamed concrete can be successfully improved by expanding the contact zone. In practice, this could be realized through the application of textile [44] or mesh reinforcements [45,46], in which a perpendicular thread provides anchorage for the fibers in the direction of the internal forces. This concept has been applied for many years to thin-walled concrete structures; it is called ferrocement, and was developed five years before the reinforced concrete.

In this paper, we develop the concept of reinforcing discussed above. Tests were carried out on 18 sets of prisms made of foamed fly-ash-based-geopolymer, reinforced with glass fiber mesh. The aim was to assess the impact of the bulk density on the mechanical properties of the foamed geopolymer and the flexural behavior of beams strengthened with GFRP (glass-fiber-reinforced polymer). The parameters investigated here included changes in the content of foaming agent (1%, 2%, and 3%), the origin of the fly ash, and localization of the reinforcement (none, external, and internal).

## 2. Experimental Program

**Figure 1:**
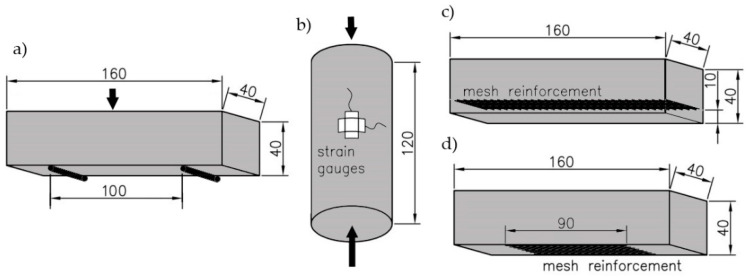
Dimensions of the sample and location of reinforcement: (**a**) beam with internal reinforcement; (**b**) beam with bottom reinforcement; (**c**) cylinder with strain sensors; (**d**) beam with bottom reinforcement. Unit: mm.

Our research program was divided into two parts. The first involved material research, and the goal was to determine the mechanical properties of the geopolymers by carrying out testing on cylinders and beams. To assess the compressive and flexural strength, prismatic beams (40 × 40 × 160 mm^3^) were cast according to EN 196-1:2016 [47], and for each mixture, three cylinders (60 × 120 mm^2^) were simultaneously made to test the modulus of elasticity and cylindrical compressive strength (Figure 1a,b).

Using the same mixture, glass-mesh-reinforced beams were made for bending resistance tests. Figure 1c,d show the dimensions of the beams and the location of the reinforcing mesh. The meshes were arranged so that each beam contained eight bundles of fibers.

The applications of glass fiber meshes in geopolymers have not yet been studied. Based on an analogy to OPC, there may be a danger that the penetration of alkaline solutions can severely damage glass fiber in terms of a loss of toughness and strength, and embrittlement [48] (p. 160). On the other hand, fiberglass has a beneficial chemical composition [49], and most studies show that dispersed glass fibers are effective in increasing the strength of a geopolymer without giving rise to embrittlement problems [50,51]. To ensure resistance to alkalis, a fiberglass lathing mesh was used. This was originally designed for cement plaster reinforcement, and is based on C-glass fiber impregnated with an alkali-resistant dispersion. The mesh size was 4.5 × 5 mm^2^, and the weight was 160 g/m^2^. The breaking strength was 25 kN/m.

### 2.1. Materials and Methods

The foamed geopolymers tested here were synthesized from fly ash, drawn from the three largest coal-fired power plants in Poland: the Jaworzno power plant, which is fired with anthracite coal, and the Belchatow and Turow power plants, which are fired with lignite coal. The chemical compositions of the fly ash materials were identified by X-ray photoelectron spectroscopy (Thermo Scientific™, Waltham, MA, USA) (using the K-Alpha™ X-ray Photoelectron Spectrometer XPS System), and are shown in Table 1.

The densities of the coal fly ashes were determined based on the EN 1097-6 [52] standard, and are presented in Table 2. The results indicate that fly ashes from the incineration of lignite coal have a higher density than those from anthracite coal.

An activator based on a sodium silicate solution (Na_2_O 8.6%, SiO_2_ 27.8%, water 63.2%) and 10 M sodium hydroxide was used. The components of the activator were mixed before use. The mass ratio of fly ash/sodium silicate/sodium hydroxide was constant for all mixtures, and was equal to 3/1.5/1 (Table 3). The mixture proportions were optimized in the strength tests of samples based on a not-foamed geopolymers.

The geopolymers were foamed by the addition of a 30% solution of hydrogen peroxide. To vary the densities of the geopolymers, three different concentrations of hydrogen peroxide with respect to the precursor were used: 1%, 2%, and 3% of the total weight ratio. In an alkaline environment, hydrogen peroxide decomposes into water and oxygen [53]; the introduction of oxygen into the mixture causes it to grow in volume. The advantage of using hydrogen peroxide for foaming is the generally homogeneous distribution of the macro-pores [29] and the increase in viscosity of the geopolymer paste [54]. However, there are also disadvantages, such as a reduction in the rate of the geopolymerization process [29]. The growing process is restricted by the hardening of the sample, which makes it relatively difficult to predict the final volume.

The same production conditions were used for all samples. The mixing procedure was as follows: the alkaline activator components were mixed for two minutes, while the precursor was simultaneously poured into the mixing bowl. After adding the activator, the components were mixed for three minutes at a constant stirrer speed of 100 RPM. Finally, hydrogen peroxide was added, and the materials were mixed for a further minute. Immediately after mixing, the samples were poured into molds containing fixed reinforcing meshes. After 10 min of growing, the excess geopolymer paste was removed, and the molds were sealed and cured in a heat chamber at 40 °C for 24 h. All the specimens were then de-molded and kept at room conditions until the test.

A total of 27 types of beams were cast, each set consisting of six identical beams made from the same mix. In addition, three cylinders were made for each mix, in order to determine the strength properties.

The following system is used here to describe the types of samples. The first letter represents the origin of the ash (J-Jaworzno, T-Turow and B-Belchatow), and the content of the foaming agent is then specified (1%, 2%, and 3%). In the case of the prismatic samples, the last two letters indicate the type of reinforcement (no-pure geopolymer (Figure 1a), in–internal mesh (Figure 1c), and bt-mesh at the bottom (Figure 1d)). For example, J_2%_in represents a beam sample made of fly ash obtained from the Jaworzno Power Plant and reinforced with an internal mesh.

### 2.2. Testing

All the specimens were tested after 28 days of curing. Before testing was carried out, the bulk density of each sample was measured.

**Figure 2:**
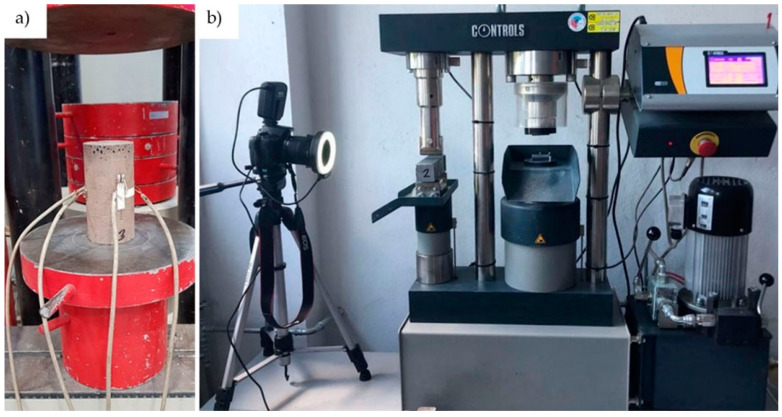
Samples during the test: (**a**) flexural test of the beam; (**b**) cylinder compressive test.

The compressive tests of the cylinders were performed using a FORM + TEST Prüffsysteme MEGA3 Testing Machine for compression testing at a loading speed of 0.1 kN/s. Strains were measured with two groups of strain gauges of length 10 mm, orthogonally adhered to the side surface of the cylinder (Figure 2a). Measurements were recorded using a Z-TECH 64-channel Wheatstone bridge.

The tests of the beams were carried out according to EN 196-1:2016, and the flexural strength was determined for each type of beam. In addition, for the pure geopolymer, compressive strength tests were done using the cube samples that remained after the bending test. The tests were performed with a Controls 65-L27C12 Universal Testing Machine at a loading speed of 0.05 kN/s. During each test, the deformation of the reinforced samples was measured using the DIC (digital image correlation) method. Pictures were taken at one-second intervals using a Canon EOS100D camera (Figure 2b). Strains and displacements were computed with GOM Correlate software.

## 3. Experimental Results and Analysis

### 3.1. Mechanical Properties of Foamed Geopolymers

The results of the cylinder tests are shown in Table 4, which gives the mean values for the three cylinders. The modulus of elasticity and Poisson’s ratio are defined as secant values within 40% of the strength.

Geopolymers synthesized from fly ash obtained from anthracite coal combustion (the Jaworzno power plant) had the best strength properties. The cylinder (uniaxial) compressive strength of geopolymer made of fly ash from Jaworzno was on average 114% higher than the values obtained for geopolymers synthesized using suspensions from lignite coal-fired power plants (Turow and Belchatow). An even greater difference was seen in the modulus of elasticity, which was on average 213% higher, and in the case of geopolymer foamed with 3% H_2_O_2_ content, up to 370% higher. These results were gained for geopolymer made from Jaworzno fly ash, despite the average lowest density in the comparison group for the same quantity of foaming agent.

**Figure 3:**
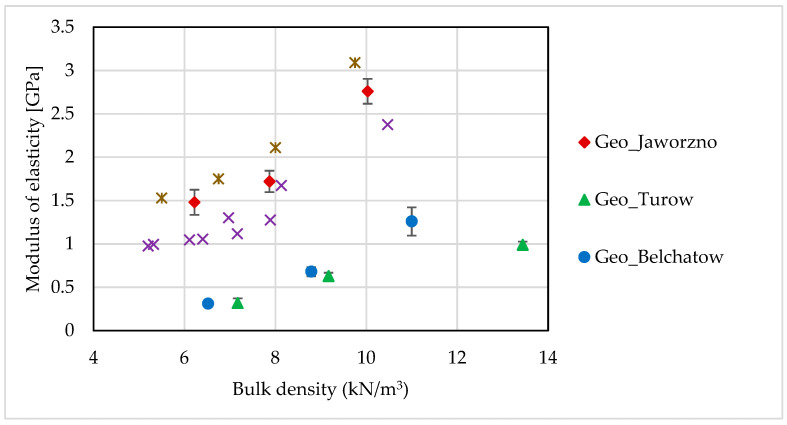
Secant modulus of elasticity vs. bulk density of tested samples: comparison with the results of foamed ordinary Portland cement (OPC) concrete tests performed by Kozlowski and Kadela [55] and Drusa et al. [56].

The modulus of elasticity for the foamed geopolymers based on fly ash from the Jaworzno power plant was comparable to those of foamed OPC concretes. Figure 3 shows a comparison with test results for foamed concrete of different densities published by Kozłowski [55] and Drusa [56].

**Figure 4:**
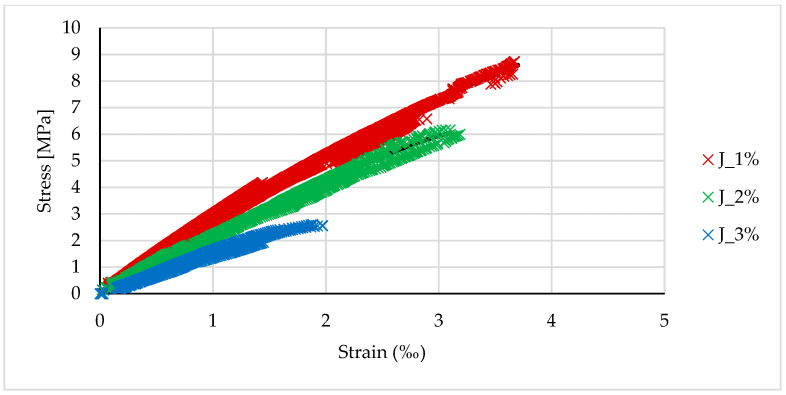
Stress-strain relationship for samples based on Jaworzno fly ash.

**Figure 5:**
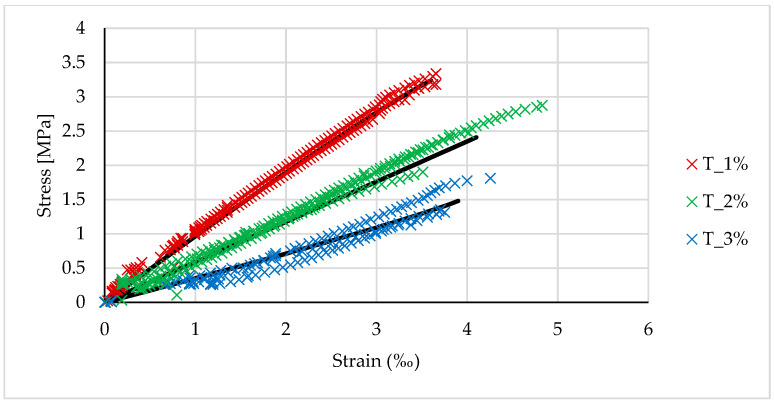
Stress-strain relationship for samples based on Turow fly ash.

**Figure 6:**
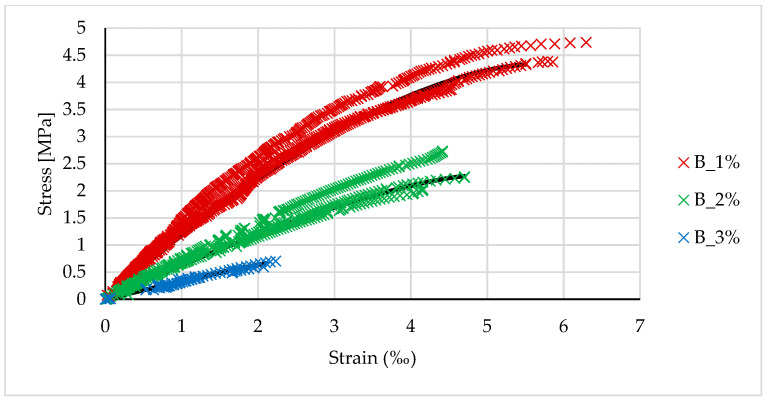
The stress-strain relationship for samples based on Belchatow fly ash.

Figure 4, Figure 5 and Figure 6 show the stress-strain relationships for geopolymers synthesized from the three different types of fly ash.

By analogy with OPC concrete, the stress-strain relationship can be described by the following function:

(1) $$\sigma =\sigma_{u}\left[1-{\left(1-\frac{\varepsilon }{\varepsilon_{u}}\right)}^{n}\right],$$

where *σ_u_* and *ε_u_* are the average strength and the corresponding ultimate strain obtained during the tests. The function in (1) was adjusted to the test results by determining the value of the power exponent *n*. The results of these analyses are summarized in Table 5. A value of *n* = 2 gives the parabolic relationship recommended for concrete, while *n* = 1 gives a linear relationship. Figure 4, Figure 5 and Figure 6 show the curves corresponding to the functions described by the parameters listed in Table 5. The fit of the functions was verified by maximizing the determination coefficient. The results obtained for R-squared are presented in the last column of Table 5.

The strengths obtained in the cylinder tests were confirmed by the beam tests, and these are summarized in Table 6, which shows the density, cube compressive strength, and tensile flexural strength. In additional, to enable further comparisons with the reinforced beams, it shows the failure force for which the flexural strength was calculated. The results were similar to those of the cylinder tests, as the average cube compressive strength was 73% higher and flexural strength 29% higher for the fly ash from Jaworzno than for those from the other two power plants.

The foaming caused a severe drop in strength. For example, the compressive strength of a plain (not foamed) geopolymer, based on fly ashes from Jaworzno, is on average 40 MPa. Strength decreases with decrease of geopolymer density. Similar relationships of strength and density were obtained in studies published by other authors [23,27]. Moreover, to a similar extent, foaming affects the strength of concretes based on Portland cement [46,55,56].

### 3.2. Efficiency of Mesh Reinforcement

Undoubtedly, the structural suitability of materials with compressive strength below 8MPa is limited to structures of secondary importance such as partition walls, leveling layers, etc. The authors’ experience shows that foamed concretes with such densities are increasingly used in load-bearing elements such as foundations, structural walls or ceilings. Usually these are composite structures with a thin concrete slab or reinforced with cores. The introduction of reinforcement should simplify such structures. The second effect of the reinforcement may be the reduction of susceptibility of usually fragile foamed geopolymer to accidental cracking.

**Figure 7:**
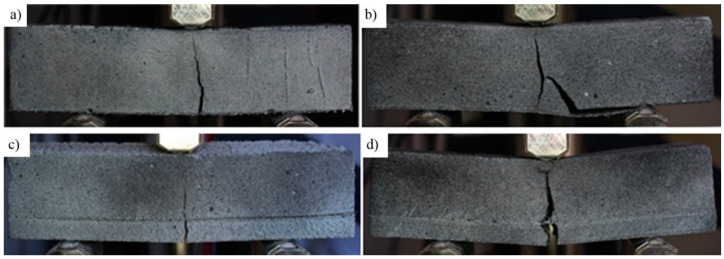
Observed failure modes: (**a**) rupture of the bottom fiber mesh; (**b**) delamination of the bottom fiber mesh; (**c**) rupture of the fiber mesh; (**d**) simultaneous crushing of concrete and fiber rupture.

The main objective of this part of research was to evaluate the effectiveness of reinforcement with glass fiber mesh. The results of these tests are presented in Table 7 and Table 8, which show the magnitude of the failure force in the bending test and the corresponding deflection, with a description of the failure mode. Three typical modes of failure were observed: rupturing of mesh fibers, delamination of the mesh, and crushing of concrete in the compressed zone, which was usually preceded by the development of flexural cracks. Examples of these are shown in Figure 7.

**Figure 8:**
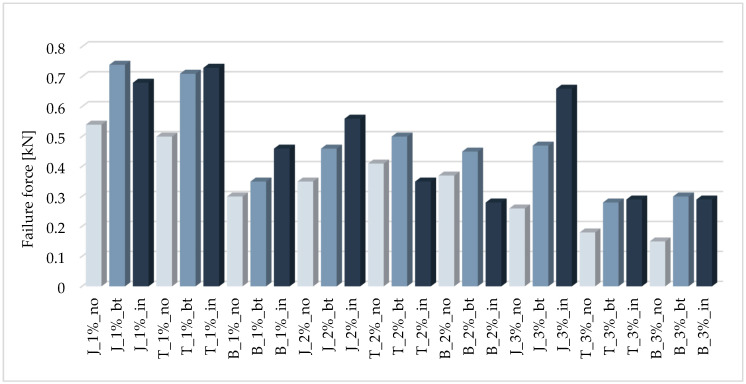
Comparison of failure forces in the bending test.

The use of reinforcement should increase the load-bearing capacity. It is clear that the reinforcement fulfilled this requirement and that the carrying capacity of the reinforced samples was greater than for models without reinforcement. The best results were obtained for the samples with the highest density, which is in line with expectations. For example, a 43% increase in flexural capacity was obtained for the T_1%_in model, and a 90% increase for the B_1%_bt model. It should be noted that all of the samples were reinforced with the same fiberglass mesh, so the greatest strengthening effects could therefore be expected for models made of the weakest material, i.e., those with the lowest density. A graphical comparison of the failure forces in the bending test is shown in the diagram in Figure 8.

Theoretically, the flexural capacity of a concrete section is most strongly influenced by the reinforcement strength and the arm of the internal forces, and the mechanical properties of the concrete itself are of secondary importance. For the models tested here, the change in load capacity after using a more foamed geopolymer was not expected to drop by more than about 12%. This hypothesis was confirmed only for the internally reinforced samples based on fly ashes from the Jaworzno power plant (J_1%_in, J_2%_in, and J_3%_in). Of the models made of C-type ash, only the T_1%_in and T_1%_bt models had a load capacity that was comparable to those based on Jaworzno fly ash.

**Figure 9:**
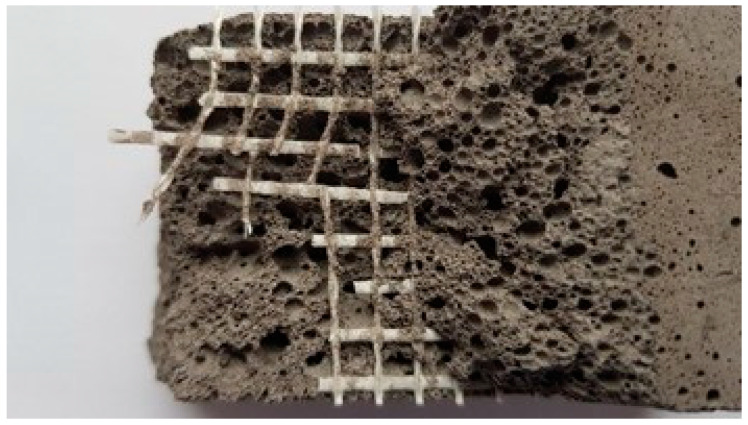
Brittle rupture of fiberglass in which the visible breakthrough does not contain free fibers.

Despite the larger arm of the internal force, the load capacity for most samples reinforced with mesh attached along the bottom surface was lower. This phenomenon can be easily explained. An analysis of the failure modes (summarized in Table 7 and illustrated in Figure 7) shows that only the densest geopolymers (foamed with 1% H_2_O_2_) provided a sufficient bond. Models foamed with additions of 2% and 3% of the foaming agent mostly broke as a result of debonding of the fiberglass mesh. The presence of pores reduces the adhesion surface, creating a poorer bond to the composite fibers, and this led to premature failure before the strength of glass fibers was exceeded. The cause of the early failure of the internally reinforced samples was different; in general, these failed after the rupture of composite fibers (Figure 7c). An inspection of the broken sample showed increased brittleness of the composite (Figure 9). The most probable reason for this is corrosion of the fiberglass caused by the alkalinity of the geopolymer. Although the tests of the samples showed a similar pH of below 11.5 for all samples, this may exceed 13.5 for a fresh mix [57]. This effect was magnified in a fresh mixture by the presence of an unreacted sodium hydroxide activator [58,59]. In the studies carried out here, all the geopolymers were fabricated using the same ratio of activator to precursor. In lignite coal fly ashes, which are poorer in aluminum and silicon, the consumption of sodium hydroxide in the reaction mechanism (geopolymer synthesis) may be slower, thus extending the exposure time of the glass mesh to the alkaline solution and causing increased brittleness of the fibers, as shown by the samples based on the Belchatow and Turow fly ashes.

**Figure 10:**
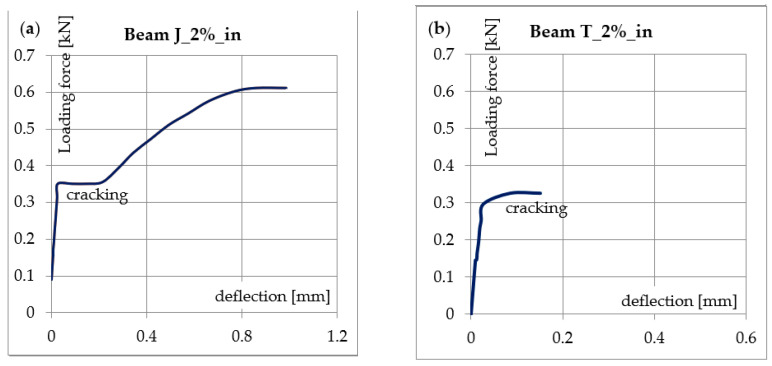
Loading force-deflection curves: (**a**) typical behavior of an internally reinforced beam; (**b**) premature failure caused by brittle fiber rupture or fiberglass debonding.

Most of the internally reinforced beams broke at deflections of between 0.5 and 1 mm, with the exceptions being the T_1%_bt and T_2%_in beams, which failed at a deflection of around 0.15 mm (Table 7 and Table 8). The reason for this specific behavior can be found by analyzing the load-deflection relationship, as shown in Figure 10. For most beams, there is an almost linear increase in the deflection in the first phase until cracking occurs (Figure 10a). There is then a rapid increase in the deflection, associated with the development of the crack, until the composite takes all the internal tensile force. We then see a further increase in the transferred load, until the fibers break. The cracking in both beams shown in Figure 10 occurred at a load of approximately 0.34 kN, and the sample shown in Figure 10b failed shortly after cracking. Premature rupture of the brittle glass fibers of the T_2%_in sample did not allow for the increase in the bearing capacity characterizing the post-crack phase, as described above. A similar effect was caused by composite delamination in the T_1%_bt sample. An increase in the brittleness of the fiberglass was noticed in all beams made of geopolymer synthesized from C-type ashes (Turow and Belchatow); however, strongly foamed material allowed for greater deflection due to crushing of the compressed zone (Figure 7d).

### 3.3. Analytical Model

**Figure 11:**
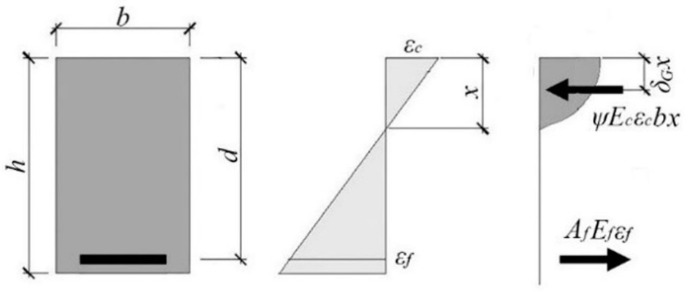
Model of a fiberglass reinforced beam under flexure.

Determination of the ultimate moment of resistance of composite-strengthened geopolymer concrete is based on similar assumptions to the calculations for steel-reinforced concrete elements. The most important are: (i) the cross-section remains a plane after deformation; (ii) the strains in composite fiber and surrounding concrete are compatible; and (iii) the tensile strength of the concrete can be ignored. Of course, due to its specific material properties, an appropriate strain limit should be assumed for geopolymers. Likewise, the stress-strain relationship must be based on a suitable function. Figure 11 illustrates the assumptions made in the model described here.

The bending resistance of a cross-section can be determined based on the equilibrium of internal forces, according to the following Equations (2) and (3):

(2) $$M_{E}=0.25F_{E}\cdot l_{b}=F_{f}(h-\delta_{G}x),$$

(3) $$\psi \varepsilon_{c}E_{c}bx=F_{f},$$

where:

$F_{f}$—fiber breaking force,
$l_{b}=100$ mm (the span of beam),
$\varepsilon_{f}$, $\varepsilon_{c}$—strains of the composite fiber and the outmost geopolymer fiber, respectively,
$E_{f}$, $E_{c}$—elasticity moduli for the composite fiber and concrete [MPa], respectively.

The lower limit of the cross-section bearing capacity is the cracking moment:

(4) $$M_{cr}=f_{ct}bh^{2}/6.$$

where $f_{ct}$ is the tensile strength of foamed geopolymer.

The plain section remains plain, therefore:

(5) $$\varepsilon_{c}=\varepsilon_{f}\frac{x}{d-x}=\frac{F_{f}}{E_{f}A_{f}}\frac{x}{d-x}.$$

As shown in Figure 4, Figure 5 and Figure 6, the stress-strain relationship can be expressed by the parabolic function in (1). In this case, the area of the compressive zone is characterized by the parameter ψ and the location of its center of gravity by the parameter $\delta_{G}$ (Figure 11). The values of these parameters change with an increase in the strain, reaching a maximum at the ultimate strain. For a linear stress-strain relationship (triangular), ψ=0.5, and δG=13. Table 9 shows the values of the coefficients ψ and $\delta_{G}$ for the geopolymers tested here, calculated for the parabolic relationship in (1) based on the assumption that the ultimate deformation of the compressed zone is reached, as shown in Table 5. The dependence of ψ and $\delta_{G}$ on the deformation significantly complicates the calculations. The parameters presented in Table 9 refer to the situation in which the ultimate strain is achieved in the compressed concrete; for lower strains, these parameters will be lower, aiming at the abovementioned values characterizing the linear relationship. The adoption of a simplified, triangular model over the entire range of deformation leads to a slight underestimate of the load capacity, which can be neglected. This is evidenced in the last column of Table 9, which shows the ratio between the capacity calculated for the triangular relationship and the capacity calculated for the parabolic Relationship (1).

For the “bt” specimens, i.e., those strengthened along the bottom surface, the force that breaks the composite fibers should not exceed the bond resistance $F_{b}$. To reflect this phenomenon, the end anchorage model was adopted (given in the *fib* bulletin 90 [60]). When the distance to the first crack $l_{b}$ is shorter than the required anchorage length, the debonding force should be limited using the formula proposed by Chen and Teng [61]:

(6) $$F_{b}\left(l_{b}\right)=F_{b}\beta_{l}=F_{b}\sin\left(\frac{\pi }{2}\frac{l_{b}}{l_{b,\lim}}\right).$$

The bond force for a fully anchored composite is equal to:

(7) $$F_{b}=0.25b\sqrt{2E_{f}t_{f}f_{c}^{2/3}},$$

and the maximum bond length, according to [60], is:

(8) $$l_{b,\lim}=0.9\pi \sqrt{\frac{E_{f}t_{f}}{8f_{c}^{2/3}}},$$

where:

$t_{f}$—thickness of composite (mm),
$f_{c}$—compressive strength of foamed geopolymer (MPa).

**Figure 12:**
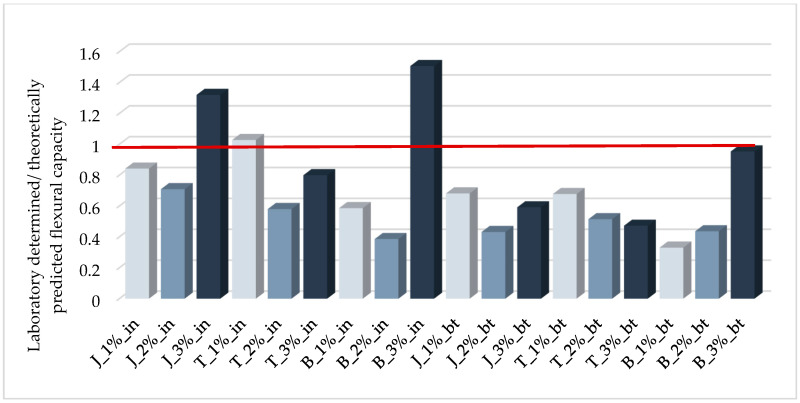
Ratios of laboratory determined/predicted flexural capacities of tested beams.

The results of a theoretical analysis of the tested beams are presented in Table 10. Figure 12 shows a comparison of these theoretical results with the values obtained from laboratory testing, expressed as a ratio of the empirical laboratory value to the result predicted using the above formulas.

An analysis of the values listed in Table 10 shows that the delamination force is greater than the fiber breaking force for the GFRP composite used here, for all models, a result that is inconsistent with the observed modes of failure (listed in Table 7). All models foamed by the addition of hydrogen peroxide >1% failed due to delamination, indicating that the delamination model adopted here significantly overestimates the results. In future, it will be necessary to develop a model that is suitable for porous materials. To the best of the authors’ knowledge, such a model has not yet been developed for externally reinforced expanded concretes.

The second reason for overestimating the load capacity in the case of internally reinforced models is the increased brittleness of the glass fibers as a result of accelerated corrosion in an alkaline environment, as described above.

**Figure 13:**
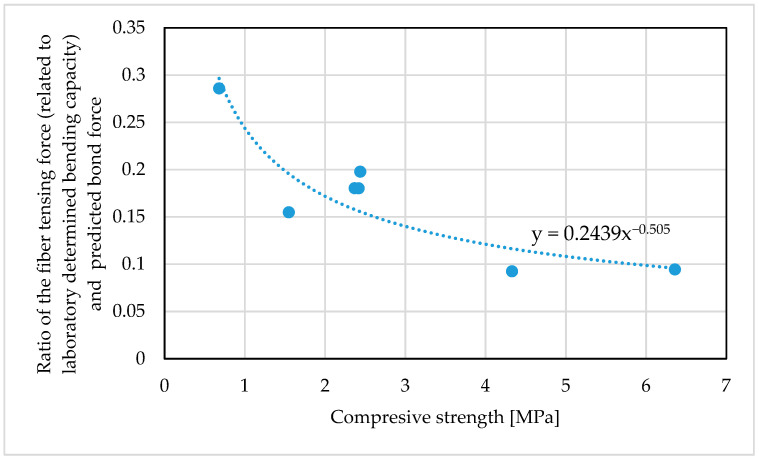
Effect of the compressive strength on the relative error of the adhesion force estimation.

The probability of premature delamination increases with the degree of foaming. The decreasing strength of the foamed geopolymer may be identified as the main factor promoting debonding. When analyzing most of the methods of predicting the debonding strength, a strong correlation between fracture energy and substrate strength can be seen [62]. For this reason, the Formula (6) can be corrected by a factor that depends on the strength of the tested geopolymer. Figure 13 shows the effect of the geopolymer strength on the relative error of the debonding force estimation (expressed as the ratio of the tensile force of the composite fiber corresponding to the laboratory-obtained bending resistance and the debonding force calculated according to Expression (6)).

The analysis of the error in estimating the debonding force enclosed the samples for which this form of failure was observed (Table 7). Relative error can be estimated with the use of the trend function shown in Figure 13. The value of this function expresses the correction coefficient of the bond force. Introducing it into (7), the modified formula for the bond to the foamed geopolymer can be obtained:

(9) $$F_{b}=0.2439f_{c}^{-0.505}0.25b\sqrt{2E_{f}t_{f}f_{c}^{2/3}}\approx 0.0625b\sqrt{2E_{f}t_{f}f_{c}^{-1/3}},$$

**Figure 14:**
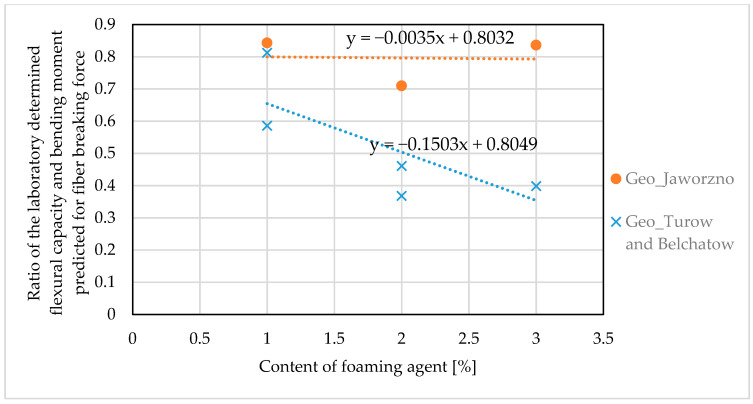
Effect of the content of foaming agent on the aging effect of glass fibers.

To find the correction factor for the corrosion aging effect, the laboratory determined and predicted flexural capacities were compared. The research showed faster corrosion in geopolymers based on lignite coal fly ash, therefore the analysis was carried out separately for samples made of ashes from Jaworzno and samples made of ashes from Turow and Belchatow. Figure 14 shows the effect of content of foaming agent on the aging factor expressed as a ratio of flexural capacity determined in laboratory and theoretically predicted for ultimate fiber breaking force (listed in Table 10).

Given in Figure 14 trend functions express the estimation error and at the same time the correction factor for to the corrosion aging effect. For the anthracite-coal-fly-ash based foamed geopolymers the fiber breaking force *F_f_* given in the Formulas (1) and (2) can be reduced according to the Equation (10):

(10) $$F_{ffg}=0.8F_{f}$$

Consequently, for the lignite-coal-fly-ash based foamed geopolymers, the reduced fiber breaking force *F_ffg_* is equal:

(11) $$F_{ffg}=(0.8-0.15D_{fa})F_{f}$$

where $D_{fa}$ is the content of foaming agent in the geopolymer mixture (given in %).

**Figure 15:**
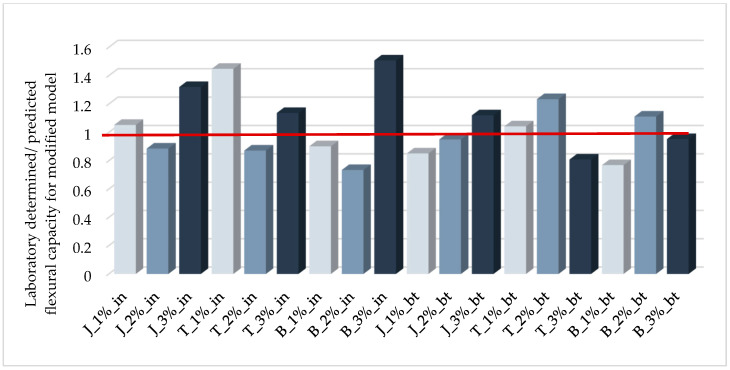
Ratios of laboratory determined/predicted flexural capacities of tested beams for empirically modified model.

To check the correctness of proposed empirically modified method, the theoretical flexural capacity of the tested samples was recalculated using the presented formulas. The results of these analyzes are shown in Figure 15. The comparison of the graphs in Figure 12 and Figure 15 shows a significant improvement in the accuracy of the load-bearing capacity estimation. The correlation coefficient for modified model is 0.835.

## 4. Conclusions

This study investigated a series of foamed geopolymer samples reinforced with fiberglass mesh. The precursors used in the synthesis of the geopolymer were fly ashes obtained from the combustion of two types of coal: anthracite and lignite. The effects of reinforcement with GFRP mesh and the type of fly ash on the strength properties of geopolymer were explored, and the results of this research and theoretical analyses give rise to the following conclusions:

(1) The origin of fly ash is of great importance for the mechanical properties of the geopolymer concrete. The compressive strengths of geopolymers based on anthracite coal fly ash are at least 73% higher than those based on lignite coal, which is rich in calcium. Similar differences were obtained in tests of the tensile strength and the modulus of elasticity. The worst mechanical properties were shown by geopolymer made from ashes from the Belchatow power plant.
(2) The deficiencies in the tensile strength can be effectively improved by reinforcement of the composite mesh. The effectiveness of this reinforcement depends on its location and the density of the foamed geopolymer. For lower densities, insufficient compressive strength leads to premature failure, which may be caused by the brittle breaking of the glass fibers or delamination.
(3) Of the beams reinforced along the bottom surface, failure due to debonding was most commonly observed. Only the samples made of fly ashes from Jaworzno and Turow and foamed with 1% H_2_O_2_ content were all broken due to mesh rupture. The proposed load capacity prediction model, which was based on existing debonding formulas originally developed for plain concrete, significantly overestimated the bond capacity.
(4) The effect of the chemical integrity of the fiberglass (Si content) [49] on improving the bond to the geopolymer was lower than expected. The suitability of glass fibers for geopolymer reinforcement, as reported by other researchers [49,50,51], was not confirmed. Chemical decomposition of the glass fibers was observed during testing, especially in the case of geopolymers based on type-C fly ashes. An increase in the brittleness of the fibers in samples inspected after testing was particularly noticeable.
(5) The proposed empirical model allows for a more precise determination of the load capacity. It takes into account the lower bond of the mesh composite to the foamed geopolymer and the aging effect of the glass fibers during the geopolymerization reaction.

Future research should focus on using reinforcing meshes that are more resistant to alkaline solutions, based on carbon or basalt fibers, and on developing a bonding model that is suitable for porous materials.

## Figures and Tables

**Table 1:** Chemical composition of fly ashes (mass %).

Power Plant	C	SiO_2_	Al_2_O_3_	CaO	SO_3_	MgO	Fe_3_O_3_	K_2_O
Jaworzno	6.87	45.04	30.88	8.13	0	1.86	4.29	2.1
Belchatow	9.18	16.65	11.15	21.40	35.58	1.87	4.16	0
Turow	8.73	18.15	13.30	22.27	29.25	2.79	4.33	0

**Table 2:** Densities of the fly ashes (kg/m^3^).

Fly Ash Origin	Jaworzno	Belchatow	Turow
Density kg/m^3^	2200	2600	2740

**Table 3:** Composition of foamed geopolymer mixtures.

Mixture	Fly Ash Origin	Fly Ash kg/m^3^	Sodium Silicate kg/m^3^	Sodium Hydroxide kg/m^3^	Hydrogen Peroxide wt %
J_1%		540.0	270	180.0	1%
J_2%	Jaworzno	403.6	201.8	134.6	2%
J_3%		327.8	163.9	109.3	3%
B_1%		601.6	300.8	200.1	1%
B_2%	Bełchatow	434.2	217.1	144.7	2%
B_3%		351.3	175.6	117.1	3%
T_1%		748.4	374.2	249.5	1%
T_2%	Turow	458.7	229.4	152.9	2%
T_3%		372.5	186.3	124.2	3%

**Table 4:** Average results of cylinder tests.

	Density kN/m^3^	Strength MPa	SD	Young Modulus GPa	SD	Poisson’s Ratio
J_1%	10.03	9.14	±0.72	2.75	±0.14	0.18
J_2%	7.78	6.36	±0.12	1.49	±0.12	0.19
J_3%	6.22	2.42	±0.28	1.48	±0.14	0.18
T_1%	13.44	4.27	±1.04	0.98	±0.036	0.18
T_2%	9.17	2.44	±0.46	0.63	±0.037	0.19
T_3%	7.17	1.55	±0.32	0.32	±0.052	0.19
B_1%	11.01	4.33	±0.43	1.26	±0.16	0.18
B_2%	8.78	2.37	±0.35	0.68	±0.053	0.18
B_3%	6.52	0.68	±0.05	0.31	±0.024	0.17

**Table 5:** Parameters for the function in (1) describing the stress-strain relationship for the foamed geopolymer.

	Strength MPa	Ultimate Strain ‰	Exponent *n*	Correlation with Test Results (R^2^)
J_1%	9.14	3.93	1.2	0.993
J_2%	6.36	3.14	1.2	0.994
J_3%	2.42	1.98	1.24	0.986
T_1%	4.27	3.64	1.09	0.996
T_2%	2.44	4.12	1	0.990
T_3%	1.55	3.96	0.9	0.964
B_1%	4.33	5.56	1.6	0.985
B_2%	2.37	4.71	1.35	0.973
B_3%	0.68	2.15	1.08	0.993

**Table 6:** Average results of beam tests.

	Density kN/m^3^	Compressive Strength GPa	Flexural Strength MPa	Ratio Flexural to Compressive	Failure Force (Bending) kN
J_1%_no	9.90	8.13	1.27	0.23	0.54
J_2%_no	7.40	3.97	0.83	0.21	0.35
J_3%_no	6.01	1.66	0.61	0.36	0.26
T_1%_no	13.72	5.26	1.17	0.22	0.50
T_2%_no	8.41	2.20	0.94	0.42	0.41
T_3%_no	6.84	1.30	0.40	0.31	0.18
B_1%_no	11.02	4.32	0.71	0.16	0.30
B_2%_no	7.96	2.25	0.85	0.37	0.37
B_3%_no	6.44	0.61	0.36	0.59	0.15

**Table 7:** Test results for beams reinforced at the bottom.

	Density kN/m^3^	Failure Force kN	Deflection at Failure mm	Failure Mode
J_1%_bt	9.93	0.74	0.41	Rupture
J_2%_bt	7.49	0.46	0.48	Rupture/delamination
J_3%_bt	6.64	0.47	0.51	Delamination
T_1%_bt	13.38	0.71	0.14	Rupture
T_2%_bt	8.42	0.50	0.36	Delamination
T_3%_bt	6.26	0.28	0.27	Delamination
B_1%_bt	11.02	0.35	0.23	Rupture/delamination
B_2%_bt	7.78	0.45	0.68	Delamination
B_3%_bt	6.08	0.30	0.48	Delamination

**Table 8:** Test results for internally reinforced beams.

	Density kN/m^3^	Failure Force kN	Deflection at Failure mm	Failure Mode
J_1%_in	10.01	0.68	0.58	Rupture
J_2%_in	7.76	0.56	1.06	Rupture
J_3%_in	6.45	0.66	1.18	Rupture/crushing
T_1%_in	13.64	0.73	0.52	Rupture
T_2%_in	9.05	0.35	0.15	Rupture
T_3%_in	6.16	0.29	0.56	Crushing/rupture
B_1%_in	11.31	0.46	0.57	Rupture
B_2%_in	8.15	0.28	0.60	Rupture
B_3%_in	6.13	0.29	0.68	Crushing/rupture

**Table 9:** Parameters for the compressed zone of the foamed geopolymer and the capacity reduction ratio for a simplified stress-strain model.

	ψ	$\delta_{G}$	Capacity Reduction Ratio for a Triangular Model
J_1%	0.545	0.344	0.953
J_2%	0.545	0.344	0.952
J_3%	0.553	0.345	0.943
T_1%	0.522	0.338	0.974
T_2%	0.500	0.333	1
T_3%	0.474	0.327	1.03
B_1%	0.615	0.361	0.887
B_2%	0.574	0.351	0.919
B_3%	0.519	0.338	0.976

**Table 10:** Comparison of laboratory determined and theoretically predicted failure forces.

	Laboratory Determined Moment Nm	Maximum Bond Length *l*_*b*,lim_ mm	Delamination Force kN	Fiber Breaking Force kN	Predicted Failure Moment Nm
J_1%_bt	18.5	96.2	4.15	0.72	27.1
J_2%_bt	11.5	105	3.30	0.72	26.6
J_3%_bt	11.7	134	1.77	0.72	19.8
T_1%_bt	17.7	116	2.55	0.72	26.1
T_2%_bt	12.5	134	1.77	0.72	24.2
T_3%_bt	7.0	150	1.32	0.72	14.8
B_1%_bt	8.75	115	2.57	0.72	26.4
B_2%_bt	11.3	134	1.75	0.72	25.7
B_3%_bt	7.5	184	0.76	0.72	7.86
J_1%_in	17.0			0.72	20.2
J_2%_in	14.0			0.72	19.7
J_3%_in	16.5			0.72	12.5
T_1%_in	18.2			0.72	17.7
T_2%_in	8.75			0.72	15.1
T_3%_in	7.25			0.72	9.06
B_1%_in	11.5			0.72	19.6
B_2%_in	7.00			0.72	18.1
B_3%_in	7.25			0.72	4.81

## Data Availability

The data presented in this study are available on request from the corresponding author.
